# Combined action of two synthetic ultrashort antimicrobial peptides exhibiting synergistic effects against clinically significant resistant bacteria

**DOI:** 10.14202/vetworld.2024.2725-2730

**Published:** 2024-12-06

**Authors:** Ali H. Salama

**Affiliations:** Department of Pharmacy, Faculty of Pharmacy, Middle East University, Amman, 11831, Jordan

**Keywords:** antimicrobial agents, bacterial infections, multidrug resistance, synergistic effects, ultra-short peptides

## Abstract

**Background and Aim:**

The emergence and proliferation of multidrug-resistant bacteria pose a global health crisis. This issue arises from the overuse and misuse of antibiotics, coupled with the pharmaceutical industry’s limited development of new drugs, which is constrained by financial disincentives and regulatory hurdles. This study aimed to investigate the combined antibacterial efficacy and safety profile of the combined ultrashort antimicrobial peptides (AMPs) WW-185 and WOW against antibiotic-resistant bacterial strains.

**Materials and Methods:**

The WW-185 and WOW peptides were synthesized through solid-phase methods and purified using reverse-phase high-performance liquid chromatography, and their purity was confirmed by mass spectrometry. Antibacterial activity was evaluated using broth dilution and checkerboard assays to assess both individual and combined effects of the peptides against *Staphylococcus aureus* (including methicillin-resistant *Staphylococcus aureus* [MRSA]) and *Escherichia coli* (including extended-spectrum beta-lactamases [ESBL]-producing strains). The synergy between the peptides was quantified using fractional inhibitory concentration indices. Hemolytic activity was also assessed to determine cytotoxicity toward red blood cells.

**Results:**

The combination of WW-185 and WOW exerted synergistic effects against both MRSA and ESBL-producing *E. coli*, with reduced minimal inhibitory concentrations compared with the individual treatments. The peptides exhibited minimal hemolytic activity, indicating low toxicity.

**Conclusion:**

The combination of the ultrashort AMPs WW-185 and WOW shows promising synergistic antibacterial effects against resistant bacteria, with potential for further therapeutic development due to their enhanced efficacy and low toxicity.

## Introduction

The spread and emergence of multidrug-resistant (MDR) bacteria represent a global health crisis. This is due to the overuse and misuse of antibiotics, compounded by the limited development of new drugs by the pharmaceutical industry, which is hindered by financial incentives and regulatory challenges [1]. To address this issue, the exploration of antimicrobial combinations has emerged as a potential solution [2]. This approach has the potential for improved effectiveness compared with single-drug therapy, as it can reduce the frequency of drug resistance and minimize the need for high-dose drugs [3].

The use of antimicrobial peptides (AMPs), a promising class of molecules, can revolutionize the treatment of bacterial and viral infections [4]. These molecules, composed of amino acids, are highly conserved across all life forms and function by disrupting the cell membranes of target organisms and inducing inflammatory responses [5]. AMPs are currently being investigated for their efficacy against various infections, including those caused by antibiotic-resistant bacteria, and their compatibility with traditional antibiotics [6]. The potential of customizable synthetic AMPs for drug development has captured the interest of researchers because of their ability to target a wide range of infections caused by antibiotic-resistant bacteria, viruses, and fungi [7]. These AMPs are typically short peptides, averaging <100 amino acids, with no conserved motifs, but are characterized by high net positive charges and elevated hydrophobic residues. Through their non-specific interactions, they can increase the permeability of negatively charged phospholipids, such as phosphatidylglycerol, which are abundant in microbial membranes, ultimately leading to cell death [8]. Despite their effectiveness in killing bacteria, several obstacles hinder the clinical use of AMPs, including poor stability *in vivo*, high manufacturing costs, low selectivity for targets, and potential toxicity [9]. To overcome these challenges, ongoing research is exploring alternative methods for designing AMPs with the goal of reducing toxicity, improving *in vivo* stability, and decreasing manufacturing costs [10]. Some strategies being investigated include hybrid peptides, combinations of different peptides, sequence modifications, and the incorporation of synthetic D-amino acids.

This study aimed to synthesize two ultrashort AMPs (USAMPs), named WOW and WW-185, and assess their individual and combined effects against clinically significant bacterial strains (*Staphylococcus aureus*, methicillin-resistant *S. aureus* [MRSA], *Escherichia coli*, and ESBL *E. coli*)

## Materials and Methods

### Ethical approval

Ethical approval was not necessary for this study.

### Study period and location

The study was conducted in Januray-2024 at the Middle East University.

### Chemicals

Mueller-Hinton Agar (Scharlab, S.L, Spain), Mueller-Hinton Broth (Oxoid LTD., England), and sterile 96-well polypropylene microtiter plates (Genetics Co. Biotechnology Products, Amman, Jordan).

### Peptide synthesis and purification

The solid-phase approach was used to synthesize and purify the two proposed peptides, which were obtained in freeze-dried form. The designated peptides used in this study were synthesized by GL Biochem Ltd. (Shanghai, China) using the Fmoc chemistry method and were ultimately obtained in a lyophilized state. For purification, reverse-phase high-performance liquid chromatography (RP-HPLC) was performed using a C18 internsil^®^ (Thermo Fisher, USA) ODS-SP column and eluted with an acetonitrile/H_2_O-TFA gradient at a flow rate of 1.0 mL/min. The purification process was confirmed by electrospray ionization mass spectrometry to identify the synthesized peptides [11, 12].

### Determining minimum inhibitory concentrations (MICs) and minimum bactericidal concentrations (MBCs) for WOW and WW-185

To determine the MIC and MBC of WOW and WW-185, the methods recommended by the Clinical and Laboratory Standards Institute [13] were followed. This involved using sterile 96-well polypropylene microtiter plates and cultivating bacterial strains in Mueller-Hinton broth (MHB) at a concentration of 10^6 colony-forming units per milliliter (CFU/mL). Dilutions of WOW and WW-185 were prepared at concentrations ranging from 0.5 μg/mL to 114.4 μg/mL. Each well in the microtiter plates contained 50 μL of each peptide, 50 μL of diluted bacterial solution, and 100 μL of MHB. Six replicates of each peptide concentration were added to the plates and incubated at 37°C for 18 h. Using an ELISA plate reader, the optical density (OD) at 570 nm was measured to evaluate bacterial growth. The MIC was defined as the lowest concentration of antimicrobial agent that inhibited visible bacterial growth. To verify bacterial growth and MHB sterility, a positive control column (50 μL bacterial suspension + 50 μL MHB) and a negative control column (100 μL MHB) were included on each plate. MBC was determined by transferring 10 μL from the clear negative well, directly following the turbid positive wells, onto sterile labeled nutrient media agar. The agar was then incubated for 24 h at 37°C. The MBC value was determined as the lowest concentration, which resulted in a 99.9% kill rate, resulting in 0.1% live cells. To ensure reliability and consistency, all experiments were performed in triplicate [14].

### Checkerboard assay

The procedure used for the assay closely mirrored that described by Jorge *et al*. [15]. For each strain, 100 μL of Mueller-Hinton broth was added to each well of the 96-well plates containing 5 × 10^^5^ CFU/mL of planktonic cells. The plates were then placed in an incubator at 37°C for 24 h, during which different concentrations (ranging from 0.025 to 114.4 μg/mL) of the two peptides were introduced. The interaction between the peptides was evaluated by calculating the fractional inhibitory concentration index (FICI) of each combination [16]. The classifications used were synergistic (FICI ≤ 0.5), additive (0.5 < FICI ≤ 1), indifferent (1 < FICI ≤ 4), or antagonistic (FICI > 4.0). This method enabled a comprehensive understanding of the combined effects and potential interactions of the peptides against the planktonic cells of the bacterial strains being studied [17].

### Hemolytic assay

The method for the hemolysis assay was adapted from Monteiro *et al*. Initially, sheep blood that had been defibrinated (100 mL; <2 weeks old; Hardy Diagnostics) was centrifuged (500× *g* at 4°C for 15 min), after which it was mixed with 10 mL of PBS. This washing procedure was repeated 3 times, and the red blood cells (RBCs) were counted using a hemocytometer. The RBCs were then diluted to a concentration of 1 × 10^9 RBC/mL in PBS and added in 100 μL amounts to each well of a 96-well white polystyrene plate (Thermo Fisher Scientific,). The plate was centrifuged at 500× *g* at 4°C for 10 min, and the supernatant was removed from all wells. Next, 100 μL of the treatment solution was added to the RBC pellet. PBS (100 μL) was used to obtain a negative control, and a positive control was created by adding 0.1% Triton X-100 (100 μL). The cells were then incubated at 37°C for 1 h and centrifuged again at 500× *g* at 4°C for 10 min. The supernatant was transferred to a 96-well clear plate with a flat bottom, and the optical density at 450 nm (OD450) was measured using a Tecan Spark plate reader. To make the readings consistent, the following equation [18] was used to calculate the percentage of hemolysis in each well. Three independent experiments were performed in duplicate [19].

## Results

### Peptide design, synthesis, and purification

**Figure-1:**
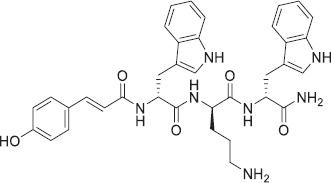
Structure of WOW peptide.

Incorporating two tryptophan subunits and one ornithine amino acid, the primary peptide WOW serves as a charge carrier, leveraging the unique benefits of an unnatural and non-coded amino acid for remarkable protease stability. Due to its effective interfacial interactions and high hydrophobicity, tryptophan is selected over other hydrophobic amino acids. Our custom-designed peptide was linked to para-hydroxycinnamic acid (PHCA) to further enhance its hydrophobicity. Notably, PHCA also possesses inherent antimicrobial properties, which are expected to enhance the overall activity of the peptide. The structure of the peptide is presented in Figure-1.

**Figure-2:**
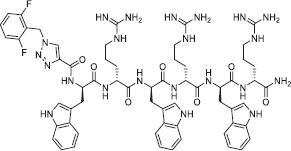
Structure of WW-185 peptide.

WW-185, also known as the second compound, is a novel and improved form of tri-AMP. The peptide comprises two units of tryptophan and one unit of ornithine, an amino acid responsible for providing charge to the peptide. One of the advantages of ornithine is that it is both an unnatural and non-coded amino acid, making it highly stable against proteases. Tryptophan, on the other hand, is incorporated into the peptide because it interacts well with the membrane interface because of its hydrophobic nature. Compared with other hydrophobic amino acids, tryptophan exhibits a strong preference. To further enhance the hydrophobic properties of the peptide, we designed a conjugation of PHCA. This not only increases hydrophobicity but also PHCA possesses antimicrobial activity. This feature is expected to increase the overall activity of the peptide. A visual representation of the peptide structure is presented in Figure-2.

### Antimicrobial activity of the two peptides

As shown in Table-1, WOW demonstrated significant effectiveness against Gram-positive *S. aureus*, with a MIC of 25 μg/mL for the standard strain and 35 μg/mL for MRSA. In contrast, for Gram-negative bacteria, the MIC was 30 μg/mL for the standard strain of *E. coli* and 50 μg/mL for ESBL. The MBC values followed a similar pattern for all four bacterial types. Shifting the focus to the second peptide WW-185, the MIC and MBC against Gram-positive bacteria were 20 μg/mL for the standard strain *S. aureus* and 25 μg/mL for MRSA. Conversely, for Gram-negative bacteria, the MIC was 35 μg/mL for *E. coli*, and the MBC was 55 μg/mL for ESBL *E. coli*.

**Table-1:** MIC and MBC values of WOW and WW-185 against the bacterial strains used.

Bacteria strains	ATCC	WOW		WW-185	
	
		MIC value (μg/mL)	MBC value (μg/mL)	MIC value (μg/mL)	MBC value (μg/mL)
*S. aureus*	25923	25	25	20	20
MRSA	BAA-2313	35	35	25	25
*E. coli*	25250	30	30	35	35
ESBL *E. coli*	BAA-2219	50	50	55	55

MIC=Minimum inhibitory concentration, MBC=Minimum bactericidal concentrations, *E. coli=Escherichia coli, S. aureus=Staphylococcus aureus*, MRSA=Methicillin-resistant *Staphylococcus aureus*, ESBL=Extended-spectrum beta-lactamase.

### Synergistic Activity of WOW along with WW-185

The synergistic effects of blending WOW and WW-185 were assessed using the checkerboard method to determine their combined antibacterial efficacy. The FICI indices were calculated to determine whether the combination of peptides resulted in a synergistic (FICI ≤ 0.5), additive (0.5 < FICI ≤ 1), indifferent (1 < FICI ≤ 4), or antagonistic (FICI > 4.0) effect. The findings of this analysis, which indicated an increase in antibacterial potency due to synergism, are presented in Table-2.

**Table-2:** The fraction inhibitory concentrations of the peptide combinations.

Bacteria strain	WOW MIC before combination	WOW MIC after combination treatment	WW-185 MIC before combination	WW-185 MIC after combination treatment	FICI*	Activity
*S. aureus*	25	0.053	20	0.5	0.02712	Synergistic
MRSA	35	3.6	25	5.62	0.327	Synergistic
*E. coli*	30	0.233	35	3.25	0.1006	Synergistic
ESBL *E. coli*	50	9.56	55	6.2	0.303	Synergistic

* FIC=Fraction inhibitory concentration, FICI=(MIC alapropoginine in combination/MIC alapropoginine alone) + (MIC RB-23 in combination/MIC RB-23) synergistic (FIC ≤ 0.5), additive (FIC 0.5 < FIC ≤ 1), indifferent (1 < FIC ≤ 4), or antagonist (FIC > 4). MIC=Minimum inhibitory concentration, MBC=Minimum bactericidal concentrations, *E. coli=Escherichia coli*, *S. aureus=Staphylococcus aureus*, MRSA=Methicillin-resistant *Staphylococcus aureus*, ESBL=Extended-spectrum beta-lactamases.

### Hemolytic activity of the peptides

The percentages of viable RBCs of both peptides are presented in Table-3.

**Table-3:** Red blood cell viable cells of WOW plus WW-185, WOW alone, and WW-185 alone.

Concentration (μM)	Hemolysis %		

	WOW peptide	WW-185	WOW combined with the WW-185
5	90	0	0
10	90	0	0
20	92	0	0
30	93	0	0
40	95	1	3
60	95	1	3
80	98	1	4
100	99	2	5

## Discussion

AMPs have emerged as promising alternatives to conventional antibiotics, especially despite increasing MDR bacterial strains. Their unique mechanism of action, which typically involves disrupting the bacterial cell membrane, provides an advantage over traditional antibiotics that target specific proteins or processes within bacteria, leading to resistance over time. A recent study by Zasloff [20] has highlighted the effectiveness of synthetic AMPs in overcoming these challenges by offering broad-spectrum activity and a low propensity for inducing resistance. In this study, we introduced two novel USAMPs, WW-185 and WOW and investigated their combined antibacterial efficacy. Our findings demonstrate the significant synergistic effects of these peptides when used together, providing a new potential therapeutic strategy for treating infections caused by resistant bacterial strains such as MRSA and ESBL-producing *E. coli*.

The combined use of WW-185 and WOW peptides resulted in marked synergism, with a FICI of ≤0.5 for all tested bacterial strains. This synergy reduced minimal inhibitory concentrations (MICs) compared with the individual peptides, suggesting that the combination is more effective at lower doses. Synergy in antimicrobial combinations is crucial because it can prevent the development of resistance by simultaneously targeting bacteria through multiple mechanisms of action [21]. In the case of WW-185 and WOW, their complementary membrane-disrupting activities likely account for the enhanced antibacterial effect observed. Combining AMPs has proven effective in reducing the required dosages of both peptides, lowering potential cytotoxicity, and enhancing antimicrobial efficacy [22].

Our findings are consistent with a study by Zasloff [20], who reported improved efficacy of antimicrobial agents when used in combination. For instance, a study by Haney *et al*. [23] has shown that combining AMPs with other agents, such as nanoparticles, can significantly enhance their antibacterial activity while potentially reducing their toxicity. However, our approach focuses on peptide-only combinations, avoiding the complications associated with nanoparticle-based therapies, which often raise concerns about long-term toxicity and environmental impact [24]. The ability of WW-185 and WOW to act synergistically suggests their potential as a combination therapy to combat MDR pathogens more effectively than single agents.

A significant challenge in developing AMPs for clinical use is their potential cytotoxicity, particularly hemolytic activity, which can damage erythrocytes. However, our study showed that the combination of WW-185 and WOW exhibited minimal hemolytic activity, indicating a favorable safety profile. Hemolysis assays revealed that combining these peptides reduced their cytotoxicity compared to individual peptides’ effects. This is a promising finding because it suggests that the combined peptides may be safer for therapeutic use, reducing the risk of harmful side effects often associated with high-dose antimicrobial treatments [25].

In the context of existing research, our approach is a significant departure from studies that have explored the use of nanoparticles to enhance the efficacy of AMPs. For instance, a study by Huang *et al*. [26], examining AMP-nanoparticle conjugates has demonstrated improved peptide stability and antimicrobial activity, but at the cost of increased toxicity and complexity in the formulation. In contrast, our study eliminated the use of nanoparticles, focusing on a simpler, peptide-only combination therapy that demonstrates comparable efficacy and circumvents the potential toxicological challenges associated with nanoparticle use. The lower toxicity observed in our study supports the idea that peptide-peptide combinations may provide a cleaner and safer therapeutic option, particularly for clinical applications.

Moreover, the combination of WW-185 and WOW offers advantages beyond enhanced efficacy. By lowering the necessary concentrations of both peptides, the synergistic interaction reduces the risk of developing resistance, a major concern in current antibiotic therapy [27]. Single-agent treatments often promote resistance by applying selective pressure on bacteria, whereas combination therapies can reduce this pressure by attacking bacterial cells through multiple pathways [28]. This approach is in line with recent trends in antimicrobial research, in which the focus has shifted toward developing combination therapies to extend the lifespan of existing antimicrobial agents and mitigate the rise of resistant strains [29].

The reduced MICs for MRSA and ESBL-producing *E. coli* in our study underscore the potential of this peptide combination to tackle difficult-to-treat infections. Resistant strains, such as MRSA and ESBL-producing *E. coli*, are particularly challenging because of their ability to evade many antibiotics currently in clinical use [30]. Our findings show that the combined use of WW-185 and WOW not only increases bacterial susceptibility and but also suggests the potential for reducing the dosage of each peptide, thus lowering the risk of toxicity and adverse side effects. This is particularly relevant for therapeutic applications in which balancing efficacy and safety is critical.

The broader implications of our study suggest that the WW-185 and WOW peptide combination could be developed as a therapeutic alternative to traditional antibiotics, especially in the fight against MDR bacteria. The strong efficacy of the peptides against both Gram-positive and Gram-negative bacteria further enhances their potential as broad-spectrum antimicrobials. However, although the *in vitro* results are promising, further research is needed to validate these findings *in vivo*. Animal models should be used to evaluate the pharmacokinetics, biodistribution, and long-term safety of the peptide combination and its efficacy against systemic infections. In addition, the potential for modification or optimization of these peptides to enhance stability and prolong their half-lives *in vivo* is a key area for future investigation.

## Conclusion

The suggestion put forth by this research is that combining WOW and WW-185 could present a more attractive option compared with conventional antibiotics in the fight against MDR bacteria. The observed synergistic effect of the combination may lead to a decrease in the necessary dosage of both peptides, resulting in reduced toxicity and improved effectiveness against bacterial infections.

## Author’s Contributions

AHS: Conceptualization and supervision, investigation, methodology, and writing–review and editing. The author has read and approved the final manuscript.
